# Telmisartan, an Antagonist of Angiotensin II Receptors, Accentuates Voltage-Gated Na^+^ Currents and Hippocampal Neuronal Excitability

**DOI:** 10.3389/fnins.2020.00902

**Published:** 2020-09-04

**Authors:** Ming-Chi Lai, Sheng-Nan Wu, Chin-Wei Huang

**Affiliations:** ^1^Department of Pediatrics, Chi-Mei Medical Center, Tainan, Taiwan; ^2^Department of Physiology, College of Medicine, National Cheng Kung University, Tainan, Taiwan; ^3^Department of Neurology, National Cheng Kung University Hospital, College of Medicine, National Cheng Kung University, Tainan, Taiwan

**Keywords:** telmisartan, voltage-gated Na^+^ current, seizure, pilocarpine, hippocampus

## Abstract

Telmisartan (TEL), a non-peptide blocker of the angiotensin II type 1 receptor, is a widely used antihypertensive agent. Nevertheless, its neuronal ionic effects and how they potentially affect neuronal network excitability remain largely unclear. With the aid of patch-clamp technology, the effects of TEL on membrane ion currents present in hippocampal neurons (mHippoE-14 cells) were investigated. For additional characterization of the effects of TEL on hippocampal neuronal excitability, we undertook *in vivo* studies on Sprague Dawley (SD) rats using pilocarpine-induced seizure modeling, a hippocampal histopathological analysis, and inhibitory avoidance testing. In these hippocampal neurons, TEL increased the peak amplitude of *I*_*Na*_, with a concomitant decline in the current inactivation rate. The TEL concentration dependently enhanced the peak amplitude of depolarization-elicited *I*_*Na*_ and lessened the inactivation rate of *I*_*Na*_. By comparison, TEL was more efficacious in stimulating the peak *I*_*Na*_ and in prolonging the inactivation time course of this current than tefluthrin or (-)-epicatechin-3-gallate. In the continued presence of pioglitazone, the TEL-perturbed stimulation of *I*_*Na*_ remained effective. In addition, cell exposure to TEL shifted the steady-state inactivation *I*_*Na*_ curve to fewer negative potentials with no perturbations of the slope factor. Unlike chlorotoxin, either ranolazine, eugenol, or KMUP-1 reversed TEL-mediated increases in the strength of non-inactivating *I*_*Na*_. In the cell-attached voltage-clamp recordings, TEL shortened the latency in the generation of action currents. Meanwhile, TEL increased the peak *I*_*Na*_, with a concurrent decrease in current inactivation in HEKT293T cells expressing *SCN5A*. Furthermore, although TEL did not aggravate pilocarpine-induced chronic seizures and tended to preserve cognitive performance, it significantly accentuated hippocampal mossy fiber sprouting. Collectively, TEL stimulation of peak *I*_*Na*_ in combination with an apparent retardation in current inactivation could be an important mechanism through which hippocampal neuronal excitability is increased, and hippocampal network excitability is accentuated following status epilepticus, suggesting further attention to this finding.

## Introduction

Telmisartan (TEL) is recognized as a non-peptide, orally active blocker of the AT1 receptor. It is a newer drug class available for the treatment of hypertension and various cardiovascular disorders (Yusuf et al., 2008; Farsang, 2011). Because TEL has a unique aromatic group that is modified, it has good lipophilicity and can hence readily penetrate the central nervous system (Stangier et al., 2000). In addition, owing to activation of PPAR-γ activity (Benson et al., 2004; Villapol et al., 2015), this compound has been found to exert anti-inflammatory actions (Benigni et al., 2010; Balaji et al., 2015; Kono et al., 2015).

In addition to the blockade of AT1 receptors and activation of PPAR-γ, TEL may be a notable regulator of membrane ion channels. For example, losartan, a prototype of the ANG II receptor antagonist, was previously found to modify cardiac-delayed rectifier K^+^ currents (Caballero et al., 2000). TEL was recently found to suppress hK_*V*_1.5 and HERG K^+^ channels functionally expressed in *Xenopus* oocytes (Tu et al., 2008). An earlier work also showed the effectiveness of TEL in retarding the inactivation of *I*_*Na*_ in rat cardiomyocytes (Kim et al., 2012).

Na_*V*_ channels are essential for the generation and propagation of APs in excitable membranes. The Na^+^ channel protein contains four homologous domains (D1–D4), each with six transmembrane segments (S1–S6). Upon brief depolarization, Na^+^ channels readily go through rapid transitions from the resting (or closed) to the open state and then to the inactivated state. Genetic defects in Na^+^ channel inactivation that lead to small sustained Na^+^ currents following the occurrence of AP firing have been recognized to have devastating consequences, including seizures, periodic paralysis, neuropathic pain, and LQT-3 syndrome (Wu et al., 2011; George et al., 2012; Jukiè et al., 2014; Qureshi et al., 2015). Nine different isoforms (Na_*V*_1.1–Na_*V*_1.9) have been found among excitable mammalian tissues, including the central nervous system, the peripheral nervous system, skeletal muscle, and heart tissues (Catterall et al., 2005). It is worth noting that most therapeutic Na^+^ channel blockers have been recognized as not isoform selective and may have more than one clinical application (Jukiè et al., 2014). Whether Na_*V*_ channels are important targets for the action of TEL remains largely unclear.

Whether the brain renin–angiotensin system can mediate seizure susceptibility also remains uncertain. Angiotensin peptides such as ang II, III, and IV have been found to have anticonvulsant properties in some seizure models (Tchekalarova and Georgiev, 2005). An intriguing study reported that TEL could have antiepileptic activities in a dose-dependent manner, as compared to olmesartan (Pushpa et al., 2014). Another report showed that TEL failed to influence the threshold for maximal electroshock-induced seizures, but it potentiated the anticonvulsant activity of valproate (Łukawski et al., 2010). However, additional previous observations indicated that the effects of captopril significantly raised the pentylenetetrazole threshold, but TEL was not shown to have this effect (Łukawski and Czuczwar, 2015). Furthermore, another study found that TEL did not provide additional anticonvulsant activity to antiepileptic drugs and that their combinations led to neurotoxic effects in animals (Łukawski et al., 2013, 2015).

It has been observed that TEL ameliorates impaired cognitive functions (Mogi et al., 2008; Du et al., 2014; Haruyama et al., 2014) and is beneficial for traumatic or ischemic brain injuries (Łukawski et al., 2014; Wang et al., 2014; Kono et al., 2015; Lin et al., 2015; Villapol et al., 2015) although the underlying mechanism has not been fully elucidated. To what extent this compound perturbs ion-channel activity and neuronal excitability in hippocampal neurons remains largely unclear. Therefore, this work was aimed toward an investigation of the *in vitro* effect of TEL on *I*_*Na*_ on a novel hippocampal neuron model and the *in vivo* effects on pilocarpine-induced seizure modeling and inhibitory avoidance in Sprague Dawley rats.

## Materials and Methods

### Cell Preparations

The embryonic mouse hippocampal cell line (mHippoE-14; CLU198) was acquired from Cedarlane CELLutions Biosystems Inc. (Burlington, Ontario, Canada) (9). Cells were grown as a monolayer culture in 50-ml plastic culture flasks in a humidifier environment comprising 5% CO_2_/95% air at 37°C. They were maintained at a density of 10^6^/ml in 5 ml of Dulbecco’s modified Eagle’s medium along with the addition of 10% heat-inactivated fetal bovine serum (v/v) and 2 mM L-glutamine. The medium was refreshed every 2 days to maintain a healthy cell population. The presence of neuritis and varicosities during cell preparation was observed. The patch clamp measurements were undertaken 5 or 6 days after the cells had undergone subculturing (60–80% confluence).

### Electrophysiological Measurements

Mouse hippocampal neurons (mHippoE-14) were harvested with 1% trypsin/ethylenediaminetetraacetic acid (EDTA) solution prior to each experiment, and a portion of the detached cells was thereafter transferred to a recording chamber mounted on the stage of a CKX-41 inverted fluorescent microscope (Olympus, Tokyo, Japan), which was coupled to a digital video system (DCR-TRV30; Sony, Japan) with a magnification of up to 1,500×. They were immersed at room temperature (20–25°C) in normal Tyrode’s solution containing 1.8 mM CaCl_2_. Patch pipettes were made from Kimax-51 glass capillaries (#34500; Kimble, Vineland, NJ, United States) using either a PP-830 electrode puller (Narishige, Tokyo, Japan) or a P-97 micropipette puller (Sutter, Novato, CA, United States), the tips of which were fire polished with an MF-83 micro forge (Narishige). The recording pipettes had a resistance of 3–5 MΩ when immersed in the different internal solutions described above. Patch-clamp recordings were made of whole-cell, cell-attached, or inside-out variants by means of either an RK-400 amplifier (Bio-Logic, Claix, France) or an Axopatch 200B amplifier (Molecular Devices, Sunnyvale, CA, United States) (14). The liquid junctional potential was adjusted immediately before sealing.

### Data Recording and Analyses

The signals were displayed on a liquid crystal projector (PJ550-2; ViewSonic, Walnut, CA, United States) and stored online in a TravelMate-6253 laptop computer (Acer, Taipei, Taiwan) at 10 kHz through a Digidata-1322A interface (Molecular Devices). The latter device was equipped with an Adaptec SlimSCSI card (Milpitas, CA, United States) via a PCMCIA card slot and controlled with pCLAMP 9.2 software (Molecular Devices). In some sets of experiments, we acquired the data by using a PowerLab acquisition system with LabChart 7.0 software (AD Instruments; Gerin, Tainan, Taiwan). Ion currents were low-pass filtered at 1–3 kHz. The signals collected during the whole-cell or single-channel experiments were analyzed offline using pCLAMP 9.2 (Molecular Devices), Origin 8.0 (OriginLab, Northampton, MA, United States) or custom-made macros built in an Excel 2013 spreadsheet running on Windows 8 (Microsoft, Redmond, WA, United States). To determine the *I–V* relationships and the steady-state activation or inactivation curves for the ion currents (e.g., *I*_*Na*_), a family of rectangular or ramp voltage pulses generated with pCLAMP 9.2 was specifically designed.

To calculate the incremental concentration-dependent effects of TEL on the peak amplitude of *I*_*Na*_ in mHippoE-14 neurons, each cell was voltage clamped at −80 mV. A brief pulse from −80 to 0 mV was delivered, and peak amplitudes were measured during exposure to TEL (0.1–100 μM). The peak *I*_*Na*_ amplitude in the presence of 100 μM TEL was taken as 100%, and current amplitudes at different concentrations of TEL were then compared to those caused by the presence of 100 μM TEL. The concentration needed to simulate 50% of the current amplitude was then evaluated by use of the Hill function. That is,

(1) $$Percentageincrease\left(\%\right)=\frac{E_{\max}+{\left[C\right]}_{50}^{n_{H}}}{{\left[C\right]}_{50}^{n_{H}}+E\,C_{50}^{n_{H^{\prime }}}}$$

where [C] is the concentration of TEL; *E*_*max*_ is the maximal increase in f peak *I*_*Na*_ caused by TEL; EC_50_ is the concentration required for 50% stimulation; and *n*_*H*_ is the Hill coefficient.

The stimulatory effect of TEL on *I*_*Na*_ can be explained by a state-dependent agonism where it binds to the open and/or inactivated state of Na_*V*_ channels according a minimal kinetic scheme given by:

(2) $$\text{Closed}\overset{\text{α}}{\underset{\text{β}}{⇄}}\text{Open}\overset{k_{+1}[\text{TEL}]}{\underset{k_{-1}}{⇄}}\text{Inactivated}$$

where α and β are the voltage-dependent rate constants for the opening and closing of Na_*V*_ channels; *k*_+__1_ and *k*_–__1_, those for forward and backward rates of current inactivation; and [TEL] is the TEL concentration. Closed, open, and inactivated shown in **Equation 2** correspond to the closed, open, and inactivated states, respectively.

Forward and backward rate constants, *k*_+1_ and *k*_-1_, were determined from the time constants of current decay evoked by the depolarizing pulses from −80 to −10 mV. The time constants of *I*_*Na*_ inactivation (t) in the control were determined by fitting the inactivation trajectory of each current trace with double exponential curve, while those with addition of different TEL concentrations were adequately made by a single-exponential function to minimize the sum of squared residuals (SSR) value. The rate constants could be estimated using a first-order scheme:

(3) $$\frac{1}{\Delta \,\tau }=k_{+1}\times \left[T\,E\,L\right]+k_{-1}$$

Specifically, *k*_+1_ and *k*_-1_, respectively, result from the slope and from the y-axis intercept at [TEL] = 0 of the linear regression interpolating the reciprocal time constants (i.e., 1/Δ*τ*) versus different TEL concentrations. Dt represents the difference in the inactivation time constant (τ) of *I*_*Na*_ obtained when the τ value during cell exposure to each concentration (1–20 μM) was subtracted from that in the presence of 30 μM TEL.

To characterize the steady-state inactivation curve of *I*_*Na*_ with or without addition of TEL, the two-step voltage profile was employed. The relationships between the conditioning potentials and the normalized amplitude of *I*_*Na*_ were plotted and well fit by the Boltzmann equation:

(4) $$\frac{I}{I_{\max}}=\frac{1}{1+e^{\left[\frac{\left(V-V_{1/2}\right)}{k}\right]}}$$

where *I*_*max*_ is the maximal activated *I*_*Na*_, *V* the membrane potential in mV, *V*_1__/__2_ the membrane potential for half-maximal inactivation, and *k* the slope factor of inactivation curve.

Action currents (ACs) that can represent APs were measured by means of cell-attached voltage-clamp recordings as described previously (Wu et al., 2009b; Hsu et al., 2014; Huang et al., 2015). Specifically, AC measurements were used to allow quantification of the underlying AP frequency under the condition where the intracellular contents were left intact. The AC waveform is mainly due to the capacitive current, which is shaped as the first derivative of the AP. The capacitive current, which can be measured when the cell fires an AP, appears as a brief spike in the downward direction.

The averaged results are presented as means ± SEM with the sample sizes (n) indicating the number of cells from which the results were taken. The linear or non-linear curve fitting to data sets presented here was performed by using either Microsoft Excel or Origin 8.0 (Microcal). The paired or unpaired *t*-test and one-way analysis of variance with the least significance difference method for multiple comparisons were used for statistical evaluation of the differences among the mean values. To determine the SSR as a function of EC_50_ value for stimulatory action of TEL on *I*_*Na*_, the 95% confidence interval was estimated using Fisher’s *F* distribution (Kemmer and Keller, 2010). The procedures were done using the “FINV” function and the “Solver” subroutine embedded in Microsoft Excel. The confidence assessment of best-fit parameter values (e.g., EC_50_) was thereafter made (Wu et al., 2015). Statistical analyses were made using IBM SPSS version 17 (Armonk, NY, United States). Statistical significance was determined at a *P* < 0.05.

### Drugs and Solutions

Telmisartan (Micardis; C_33_H_30_N_4_O_2_; 4′-[(1,4′-dimethyl-2′-propyl[2,6′-bi-1*H*-benzimidazol]-1′-yl)-1′-yl]methyl)-[1,1′-biph enyl]-2-carboxylic acid; TEL) was obtained from Tocris Cookson Ltd. (Bristol, UK), while aconitine (ACO), angiotensin II, (-)-epicatechin-3-gallate (ECG), eugenol (EUG), pioglitazone, tefluthrin (Tef), tetraethylammonium chloride (TEA), and tetrodotoxin (TTX) were obtained from Sigma-Aldrich (St. Louis, MO, United States). KMUP-1 was kindly provided by Dr. Bin-Nan Wu (Kaohsiung Medical University); chlorotoxin was provided by Dr. Woei-Jer Chuang (National Cheng Kung University), and all other chemicals, including CdCl_2_, CsCl, and CsOH, were commercially available and reagent grade. Double-distilled water deionized through a Millipore-Q system (Bedford, MA, United States) was used in all experiments.

The composition of the normal Tyrode’s solution was 136.5 mM NaCl, 5.4 mM KCl, 1.8 mM CaCl_2_, 0.53 mM MgCl_2_, 5.5 mM glucose, and 5.5 mM HEPES–NaOH buffer, pH 7.4. During the experiments recording K^+^ currents or membrane potential, a patch electrode was filled with a solution consisting of 140 mM KCl, 1 mM MgCl_2_, 3 μM Na_2_ATP, 0.1 mM Na_2_GTP, 0.1 mM ethylene glycol tetraacetic acid (EGTA), and 5 mM HEPES–KOH buffer at a pH of 7.2. To measure *I*_*Na*_ or *I*_*Ca,L*_, the KCl inside the pipette solution was replaced with equimolar CsCl, and the pH was then titrated to 7.2 with CsOH. To avoid possible contamination of the Cl^–^ currents, the Cl^–^ ions inside the pipette solution were replaced with aspartate.

### Animal Experiments

All experiments, including the animal experimentation procedures, were reviewed and approved by the Institutional Animal Care and Use Committee (IACUC) at National Cheng Kung University. All the institutional biosafety and biosecurity procedures were strictly adhered to. Efforts were made to reduce the number of rats used. Adult Sprague–Dawley male rats weighing 180–200 g were purchased from National Cheng Kung University. They were housed in the university’s Animal Center and allowed free access to water and a pelleted rodent diet (Richmond Standard; PMI Feeds, St. Louis, MO, United States).

### Lithium–Pilocarpine-Induced Seizure Modeling and Spontaneous Recurrent Seizures

On day 1, the rats were injected with lithium chloride (3 meq/kg; ip) and methylscopolamine (25 mg/kg; sc) and then subjected to pilocarpine (60 mg/kg; sc)-induced seizures. The behavioral characteristics of the rats during epileptic seizures were similar to those reported elsewhere (Lai et al., 2018a,b; Hung et al., 2019). During pilocarpine-induced status epilepticus, the rats were given zoletil (50 mg/kg, ip) and xylazine (20 mg, ip) and atropine (0.2 mg/kg, sc) if the status epilepticus lasted for 20 min (Mello et al., 1993). All the rats were monitored continuously for the first 24 h by two experienced research assistants after status epilepticus, and they were given supportive care: body temperature maintenance with a resistive heating system, food, and adequate hydration with normal saline (0.9% w/v of NaCl, 308 mOsm/L). Any animals showing intense signs of acute respiratory distress were immediately euthanized with a sodium pentobarbital overdose (150 mg/kg, ip). Then, the rats were divided into an experimental group (TEL) [orally fed TEL (10 mg/kg, twice daily) for 7 consecutive days] and a control group (orally fed normal saline daily for 7 consecutive days). We began monitoring spontaneous recurrent seizures 7 days after status epilepticus. The rats were monitored with a video camera mounted above the cage for 8 h/day over 5 consecutive days (Maroso et al., 2010). A trained technician blinded to the experimental design examined the videos for seizure behavior (i.e., running, jumping, rearing, lordosis, and erect tail). When seizure-like activity was observed, the video was reviewed to confirm seizure behaviors.

### Inhibitory Avoidance Task

The single-trial inhibitory avoidance (IA) task, another hippocampus-dependent behavior test, was used to measure different memory phases in the rats on day 14. The apparatus consisted of one illuminated compartment and one dark compartment. A shock generator was connected to the floor of the dark compartment. Before the experiment, the rat was kept in a dim room for 1 h to adjust to the environment. In the training phase, the rat was placed in the illuminated compartment facing away from the door. As the rat turned around, the door was opened. When the rat entered the dark compartment, the door was closed, and the rat was given a 1.0 mA/1-s shock. The reaction to the shock was graded as flinch, vocalization, or locomotion. The rat then was removed from the alley and returned to its home cage. The retention test was given 1, 3, or 24 h after training for the measurement of short-term, intermediate, and long-term memory, respectively. The rat was again placed in the illuminated compartment, and the latency prior to stepping into the dark compartment was recorded as the measure of retention performance. Rats that did not enter the dark compartment within 600 s were removed from the alley (Chang et al., 2003).

### Histopathology

#### Cresyl Violet Staining

Cresyl violet staining was used for the evaluation of neuronal loss in animals that had chronic recurrent seizures. On day 18, the rats’ brains were removed and stored at −80°C, 0.9% NaCl. Paraformaldehyde was then used for perfusion. Coronal sections (20 μm thick) of the hippocampus were fixed in formaldehyde, as previously described (Chang et al., 2003; Lai et al., 2018a,b). The cresyl violet-stained sections were examined for gross indications of damage to the hippocampus. The cells were counted in Nissl-stained sections (10 μm thick). The severity of neuron loss in different subfields of the hippocampus was scored semiquantitatively as follows: 0 = no neuron loss, 1 ≤ 10% neuron loss, 2 = between 11 and 50% neuron loss, and 3 ≥ 50% neuron loss (Pitkänen et al., 2006; Lai et al., 2018a,b; Hung et al., 2019). Counts were made at 400 × using the Image Plus 2.0 computer image analysis system (Motic, Richmond, BC, Canada), and the hippocampal subfields were defined by an imaginary line connecting the tips of the granule cell layer blades, which separated the Cornu Ammonis3c (CA3c) (medially) from the CA3b (laterally) and the CA2 from the CA1 (Chang et al., 2003; Lai et al., 2018a,b; Hung et al., 2019). Values from the different groups were determined by an investigator blind to the study design, after which they were averaged in each group.

### Timm’s Staining

To evaluate whether TEL had a chronic poststatus epilepticus effect on neuron excitability, we used Na_2_S and then paraformaldehyde for perfusion. On day 18, the rats’ brains were removed, and coronal sections (20 μm thick) through the entire hippocampus were cut on a Leica CM1900 freezing microtome. Every sixth section was stained with Timm’s stain (Chang et al., 2003; Lai et al., 2018a,b; Hung et al., 2019). The sections were developed in the dark for 10–45 min in a 200-ml solution containing 5.1 g of citric acid, 4.7 g of sodium citrate, 3.47 g of hydroquinone, 212.25 mg of AgNO_3_, and 120 ml of 50% gum arabic. Timm’s staining was assessed from the septal area to the temporal hippocampus (the region between 2.8 and 3.8 mm posterior to the bregma). We used a semiquantitative scale to evaluate the degree of mossy fiber sprouting in the pyramidal and infrapyramidal areas of the hippocampus CA3 region and that in the granular cell and inner molecular layers of the dentate gyrus (Chang et al., 2003; Lai et al., 2018a,b; Hung et al., 2019). The score criteria were as follows: 0, no granules; 1, occasional discrete granule bundles; 2, occasional-to-moderate granules; 3, prominent granules; 4, a prominent nearly continuous granule band; and 5, a continuous or nearly continuous dense granule band.

### Statistical Analysis

Values are provided as means ± standard error of the mean (SEM) with the sample sizes (n) indicating the number of cells from which the data were collected. Significance was set at *p* < 0.05. Continuous variables were assessed using *t-*tests or a one-way analysis of variance ANOVA SPSS 15.0 (SPSS Institute, Chicago, IL, United States) and then Fisher’s least significant difference tests. However, because the Shapiro–Wilk normality test showed that the data may not have been normally distributed, the Kruskal–Wallis *H* test, followed by Dunn’s multiple comparison tests, were applied. Analyses were done using χ^2^ tests, the Yates χ^2^ test, or Fisher’s exact test. Continuous data are expressed as means ± SEM unless otherwise indicated.

## Results

### Stimulatory Effect of TEL on *I*_*Na*_ in mHippoE-14 Neurons

**FIGURE 1:**
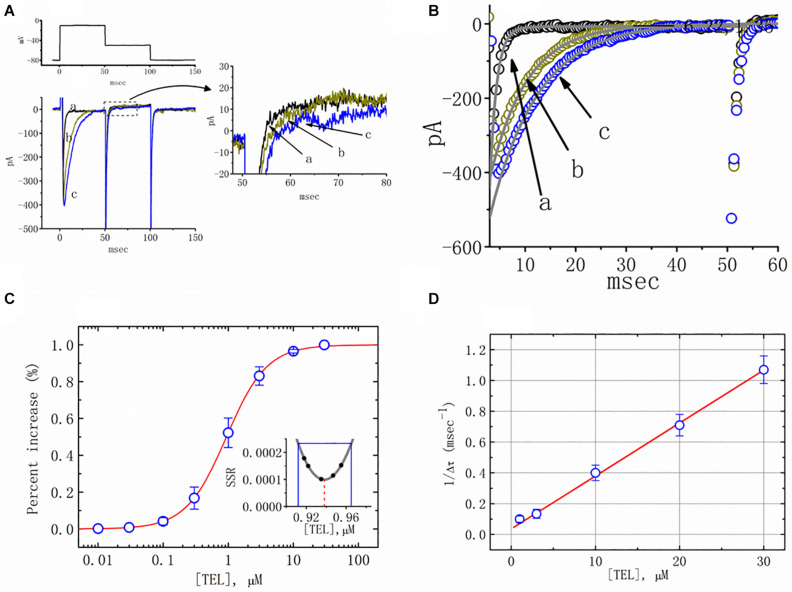
Effects of telmisartan (TEL) on voltage-gated Na^+^ current (*I*_*Na*_) in mHippoE-14 neurons. In these experiments, cells were suspended in Ca^2+^-free Tyrode’s solution containing 0.5 mM CdCl_2_ and 10 μM TEA. A pipette was filled with a Cs^+^-containing solution. **(A)** Original *I*_*Na*_ traces attained in the control (a) and in the presence of 1 μM (b) and 3 μM (c) TEL. The voltage pulses indicated in the upper part of **(A)** were depolarized from −80 to −10 mV and stepped back to −50 mV, at which point, tail currents were measured (right part). The right part of **(A)** indicates expanded records of tail currents, which correspond to the traces shown in the dashed box. **(B)** Perturbating effects of TEL on inactivation time course of *I*_*Na*_. Current trajectory for the inactivation time courses of *I*_*Na*_ in the absence or presence of TEL was well fitted by one- or two-exponential functions, respectively. a: control; b: 1 μM TEL; c: 3 μM TEL. **(C)** Concentration-dependent effect of TEL on the percentage increase in peak *I*_*Na*_ (mean ± SEM; *n* = 9-13 for each point). The smooth red line in **(C)** represents the best fit to a modified Hill equation (**Equation 1**). The EC_50_ value and Hill coefficients for TEL-induced stimulation of *I*_*Na*_ were 0.94 mM and 1.4, respectively. The inset in **(C)** shows the confidence assessment of the best-fit parameter values (i.e., EC_50_). The parameter range corresponds to an approximate 95% confidence interval. The blue line marks the parameter value at which SSR equals 0.000234, and the broken red line is clearly pointed at the EC_50_ value. **(D)** Evaluation of the kinetics of TEL-induced effects on the inactivation time course of *I*_*Na*_. The reciprocal of Dt was plotted against the TEL concentration (explained in **Equations 3** and **4**). Forward (*k*_+__1_) or backward (*k*_–__1_) rate constants, given by the slope and the y-axis intercept of the interpolated line, were found to be 0.0344 ms^–1^ μM^–1^ and 0.0368 ms^–1^, respectively. Each point is the mean ± SEM (*n* = 9–12).

In the first stage of the measurements, we bathed the cells in Ca^2+^-free Tyrode’s solution that contained 0.5 mM CdCl_2_ and 10 μM TEA. The *I*_*Na*_ was evoked in response to a brief depolarizing pulse from −80 to −10 mV, and the tail current at the level of −50 mV was measured. The peak amplitude of *I*_*Na*_ was profoundly increased as cells were exposed to TEL (Figure 1A). For example, as the depolarizing voltages from −80 to −10 mV were applied, the exposure to TEL (3 μM) noticeably elevated the peak amplitude of *I*_*Na*_ from 181 ± 19 to 398 ± 31 pA (*n* = 11, *p* < 0.05). This stimulatory effect was readily reversed following the TEL washout.

Additionally, a noticeable change in the inactivation time course of *I*_*Na*_ was seen in the presence of TEL. As the cells were exposed to TEL, inward currents were found to activate to a maximum, and the decay in response to briefly maintained depolarization slowed significantly (Figure 1B). For example, under controlled conditions, the *I*_*Na*_ elicited by step depolarization to −10 mV decayed with fast and slow time constants of 1.32 ± 0.04 and 6.31 ± 0.08 ms (*n* = 12). However, as the cells were exposed to 1 or 3 μM TEL, the inactivation time course measured at −10 mV was adequately fitted to a one-exponential with a time constant of 7.29 ± 0.12 (*n* = 8) or 9.47 ± 0.19 ms (*n* = 9), respectively. The time course of current decay (or relaxation) after returning to −50 mV was also observed to slow in the presence of TEL. These experimental observations reflect that the presence of TEL increased the amplitude of *I*_*Na*_ in a concentration- and time-dependent fashion.

The relationship between the TEL concentration and the peak *I*_*Na*_ amplitude was examined next. Each cell was depolarized from −80 to −10 mV, and the peak amplitudes at different concentrations of TEL were compared. As illustrated in Figure 1C, TEL increased the strength of peak *I*_*Na*_ in a concentration-dependent manner. The EC_50_ value for TEL-stimulated *I*_*Na*_ was found to be 0.94 ± 0.04 μM, where TEL at a concentration of 30 μM fully increased the peak amplitude of *I*_*Na*_. The SSR plot in the inset of Figure 1C shows a horizontal line at SSR = 0.000234, which was utilized to denote the two EC_50_ values. For a 95% confidence interval, the lower and upper values were 0.912 and 0.965 μM, respectively. Owing to a steep slope on both sides of the minimal SSR, the EC_50_ value for the TEL-stimulated *I*_*Na*_ could be clearly observed (Kemmer and Keller, 2010). The data thus indeed indicated that TEL exerts a stimulatory effect on the *I*_*Na*_ amplitude.

### Evaluating TEL’s Time-Dependent Attenuation of *I*_*Na*_ Inactivation

Increasing TEL concentrations not only led to increased amplitude in the peak *I*_*Na*_ but also produced a significant retardation in the strength of the *I*_*Na*_ inactivation. According to the first-order binding scheme (**Equations 2** and **3**), the relationship between 1/Δ*τ* and [TEL] became linear with a correlation coefficient of 0.98 (Figure 1D). The forward and backward rate constants were consequently calculated to be 0.0344 ms^–1^ μM^–1^ and 0.0368 ms^–1^, respectively. Owing to these resultant rate constants, the apparent dissociation constant (i.e., *K*_*D*_ = *k*_–__1_/*k*_+__1_) for the binding of TEL to Na_*V*_ channels was found to be 1.04 μM, a value that was close to the estimated EC_50_ value for TEL-perturbed stimulation of peak *I*_*Na*_ determined from the concentration–response curve elaborated above (Figure 1C).

### Comparisons of TEL, Tefluthrin (Tef), (-)-Epicatechin-3-Gallate, or Aconitine on *I*_*Na*_

**FIGURE 2:**
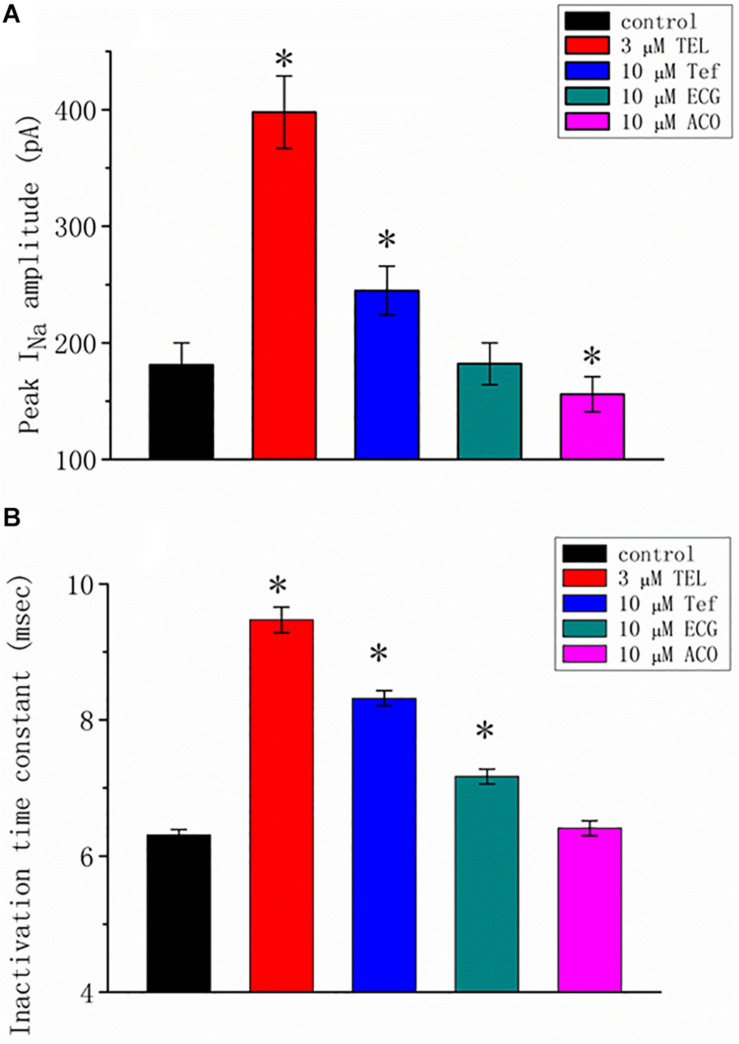
Summary of the data showing the effects of telmisartan (TEL), tefluthrin (Tef), (-)-epicatechin-3-gallate (ECG), and aconitine (ACO) on the **(A)** peak amplitude and **(B)** inactivation time constant of *I*_*Na*_ evoked by a brief depolarizing pulse ranging from −80 to −10 mV (mean SEM; *n* = 12–15 for each bar). The trajectories in *I*_*Na*_ inactivation in the presence of the various tested compounds were adequately fit using a single exponential process, while those in the control were fit using a two-exponential process. The value in the control represents the slow component of current inactivation. ^∗^Significantly different from control (*p* < 0.05).

We additionally compared the stimulatory effect of TEL on *I*_*Na*_ with the effects of Tef, ECG, and ACO. Tef, ECG, and ACO have been previously disclosed to activate *I*_*Na*_ (Fu et al., 2006; Wu et al., 2009b, 2013). *I*_*Na*_ was elicited using a depolarizing pulse ranging from −80 to −10 mV, and the peak amplitude was measured and compared as the different tested compounds were added. As shown in Figure 2, TEL at a concentration of 3 μM was more effective in terms of enhancing the amplitude of peak *I*_*Na*_ as well as of relaxing the inactivation time course of *I*_*Na*_ as compared to either Tef or ECG. However, the presence of ACO (10 μM) suppressed the peak *I*_*Na*_, with no pronounced perturbation in the inactivation time course.

### Effect of Pioglitazone and Pioglitazone Plus TEL on *I*_*Na*_

**FIGURE 3:**
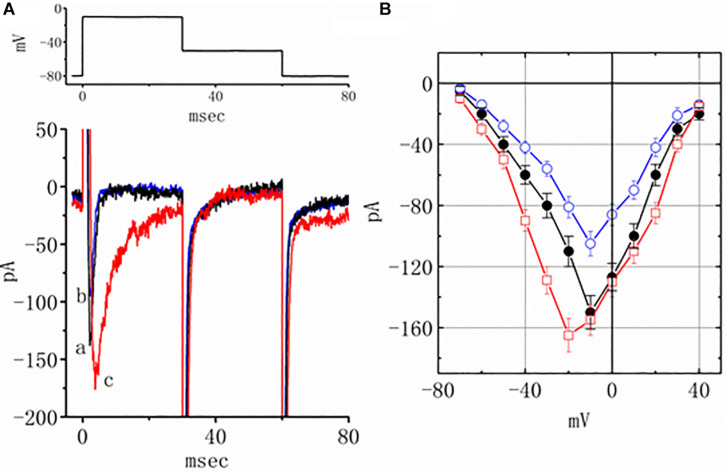
Effects of pioglitazone and pioglitazone plus telmisartan (TEL) on *I*_*Na*_ in mHippoE-14 neurons. **(A)** Original current traces attained in the control, pioglitazone, and pioglitazone plus TEL. The upper part indicates the voltage protocol used. a: control; b: pioglitazone (3 μM); c: pioglitazone (3 μM) plus TEL (3 μM). In the experiments with pioglitazone plus TEL, in the continued presence of pioglitazone (3 μM), TEL (3 μM) was subsequently applied. **(B)** *I–V* relationships of peak *I*_*Na*_ obtained in the control and in presence of pioglitazone and pioglitazone plus TEL (mean ± SEM; *n* = 7–9 for each point). ⚫: control; ◯: pioglitazone (3 μM); ☐: pioglitazone (3 μM) plus TEL (3 μM).

Earlier reports have revealed the effectiveness of TEL in modulating the activity of PPAR-γ (Benson et al., 2004; Mogi et al., 2008; Balaji et al., 2015). The effect of piogliazone, a thiazolidinedione known to activate PPAR-γ, on *I*_*Na*_ was tested in this study. As shown in Figure 3, when cells were exposed to pioglitazone (3 μM), the peak amplitude of the current measured at −10 mV was lessened from 150 ± 11 to 105 ± 8 (*n* = 8, *p* < 0.05). Neither a change in the inactivation time course nor the *I–V* relationship for this current could be observed in the presence of pioglitazone. However, in the continued presence of pioglitazone (3 mM), further addition of TEL (3 μM) enhanced the peak *I*_*Na*_ along with a profound slowing in current inactivation, as demonstrated by a significant raise in the peak *I*_*Na*_ amplitude to 155 ± 10 (*n* = 7, *p* < 0.05). The *I–V* relationships among the values of the peak *I*_*Na*_ in the absence or presence of pioglitazone and pioglitazone plus TEL were constructed, as illustrated in Figure 3B. Further addition of TEL (3 μM) caused a slight left shift in the peak *I*_*Na*_ *I–V* relationship. Therefore, the effects of pioglitazone on *I*_*Na*_ tended to be distinct from those of TEL in spite of the effectiveness of both compounds in exerting agonistic activity in PPAR-γ.

### Ability of TEL to Perturb the Peak *I*_*Na*_ *I*–*V* Relationship and the Steady-State Inactivation Curve

**FIGURE 4:**
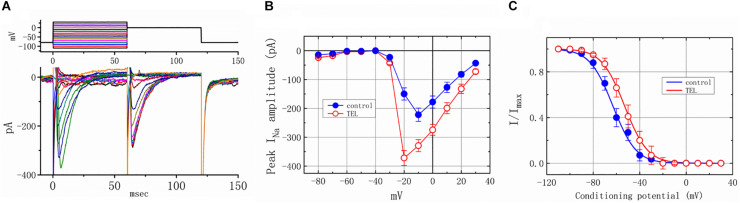
Voltage dependence of the stimulatory effect of TEL on *I*_*Na*_ in mHippoE-14 neurons. Cells were bathed in Ca^2+^-free Tyrode’s solution containing 10 μM TEA. **(A)** Superimposed voltage and current tracings in the presence of 3 μM TEL. The voltage profile tested is shown in the upper part of **(A)**. **(B)** *I–V* relationships of peak *I*_*Na*_ in the absence (⚫) and presence (◯) of 3 μM TEL (mean ± SEM; n = 8–9 for each point). **(C)** Steady-state inactivation curve of *I*_*Na*_ in the absence (⚫) and presence (○) of 3 μM TEL (mean ± SEM; *n* = 8–9 for each point). The steady-state inactivation parameters with and without the addition of Tel (3 μM) were obtained. The normalized amplitude of *I*_*Na*_ (*I*/*I*_*max*_) was constructed against the conditioning potential, and the smooth curves were well fit with the Boltzmann equation (**Equation 4**).

As shown in Figures 4A,B, the effects of TEL on *I*_*Na*_ were examined at different membrane potentials, and an *I–V* current relationship was established. The peak *I*_*Na*_ *I–V* relationship was observed to shift slightly to more negative potentials during cell exposure to TEL (3 μM). To characterize the stimulatory effect of TEL on *I*_*Na*_, we also explored whether there were any perturbations in the *I*_*Na*_ inactivation curve during cell exposure to this compound. Figures 4A,C show the steady-state *I*_*Na*_ inactivation curves obtained following the application of TEL (3 μM). The relationship between the *I*_*Na*_ conditioning potentials and normalized amplitudes were derived and well fit with a Boltzmann function (Equation 4). The resultant values for half-maximal inactivation (*V*_1__/__2_) or the corresponding slope factor (*k*) in the control were −63.1 ± 1.2 and 9.1 ± 0.2 mV (*n* = 9), respectively; however, during exposure to 3 μM TEL, the values of *V*_1__/__2_ and *k* were −52.9 ± 1.2 and 9.0 ± 0.2 mV (*n* = 8), respectively. The results reflect that the steady-state *I*_*Na*_ inactivation curve during exposure to TEL was shifted rightward, with no clear adjustment in the slope factor of this curve.

### Effect of TEL on Recovery of *I*_*Na*_ From Inactivation

**FIGURE 5:**
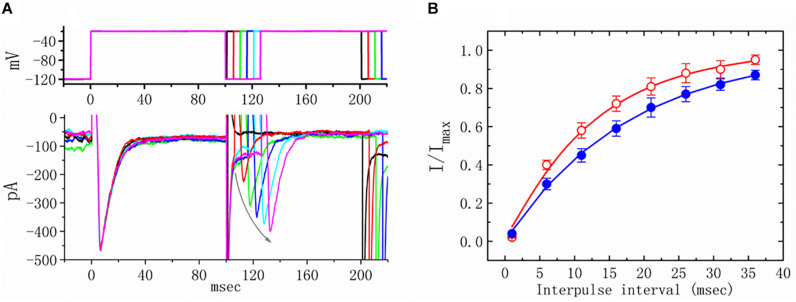
Effects of telmisartan (TEL) on the recovery of *I*_*Na*_ from inactivation in mHippoE-14 neurons. Cells bathed in Ca^2+^-free Tyrode’s solution were briefly depolarized from −120 to −20 mV with a duration of 100 ms, and various interpulse durations were applied (indicated in the upper part of panel **A**). In **(A)**, an example of current traces obtained with a two-pulse protocol in the presence of TEL (3 μM) is illustrated. Noticeably, the curved gray arrow shown in **(A)** indicates the direction of *I*_*Na*_ recovery. **(B)** Time course of recovery from inactivation of *I*_*Na*_ in the absence or presence of 3 μM TEL (mean ± SEM; *n* = 12–14 for each point). The time course in the absence or presence of TEL (3 μM) was fitted to a single exponential with a time constant of either 17.7 or 12.5 ms, respectively.

We further examined the perturbations of TEL as related to causing *I*_*Na*_ to recover from inactivation. In this two-step voltage protocol, a 100-ms conditioning potential to −20 mV inactivated most of the *I*_*Na*_, and the recovery of *I*_*Na*_ from inactivation at a holding potential of −120 mV was thereafter examined at different times with a test step (-20 mV, 100 ms), as shown in Figure 5. In the control condition (i.e., TEL was not present), the peak amplitude of *I*_*Na*_ almost fully recovered from inactivation when the recovery time was 50 ms. The time course of recovery from current inactivation was fitted to a single exponential function with a time constant of 17.7 ± 2.1 ms (*n* = 14). Alternatively, in the presence of TEL (3 μM), recovery from inactivation turned out to be faster, as demonstrated by a noticeable reduction in the time constant to 12.5 ± 1.8 ms (*n* = 12, *p* < 0.05). It was, therefore, apparent that TEL causes potential shortening of recovery from the inactivation of *I*_*Na*_.

### Enhancing the Effects of TEL on Late *I*_*Na*_ Identified in mHippoE-14 Neurons

**FIGURE 6:**
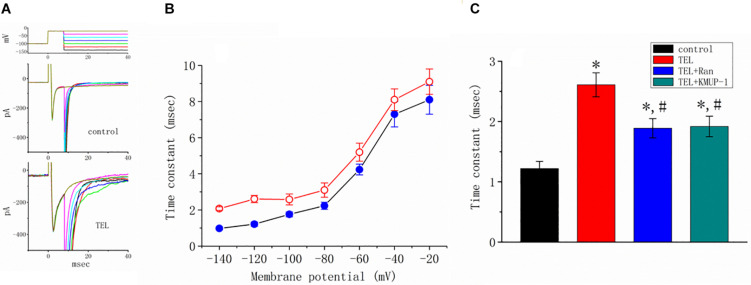
Effects of telmisartan (TEL) and TEL plus Ran on peak and late components of *I*_*Na*_ identified in mHippoE-14 neurons. The conditioning pulses were briefly depolarized from −80 to −20 mV for a duration of 8 ms. After the conditioning pulse, the membrane potential was set at different potentials ranging from −140 to −20 mV in 20-mV increments to elicit late *I*_*Na*_. **(A)** Superimposed current traces obtained in the control (upper) and during cell exposure to 3 μM TEL (lower). The upper part denotes the voltage protocol delivered. **(B)** Relationship of membrane potential versus the inactivation time constant (t) of late *I*_*Na*_ obtained in the absence (⚫) and presence (○) of 3 μM TEL (mean ± SEM; *n* = 7–9 for each point). **(C)** Summary of the data showing effects of TEL (3 μM), TEL (3 μM) plus Ran (10 μM), and TEL (3 μM) or KMUP-1 (10 μM) on the inactivation time constant of late *I*_*Na*_ (mean ± SEM; *n* = 7–9 for each bar). *Significantly different from the control (*p* < 0.05) and ^#^significantly different from the TEL (3 μM)-alone group (*p* < 0.05).

How TEL interacted with late *I*_*Na*_ to alter the time course of *I*_*Na*_ inactivation was further investigated. As depicted in Figure 6, according to another two-step voltage protocol, TEL (3 μM) not only increased peak *I*_*Na*_ but also clearly prolonged the inactivation time course of late *I*_*Na*_. More importantly, in the continued presence of TEL (3 μM), subsequent application of Ran (10 μM) or KMUP-1 (10 μM) significantly reversed TEL-perturbed increases in the inactivation time constant (t) of late *I*_*Na*_. Ran and KMUP-1 have been previously used to suppress peak *I*_*Na*_ and to elevate the *I*_*Na*_ inactivation rate (Chen et al., 2009; Lo et al., 2015).

### Stimulating Effect of TEL on the *I*_*Na(NI)*_ in mHippoE-14 Neurons

**FIGURE 7:**
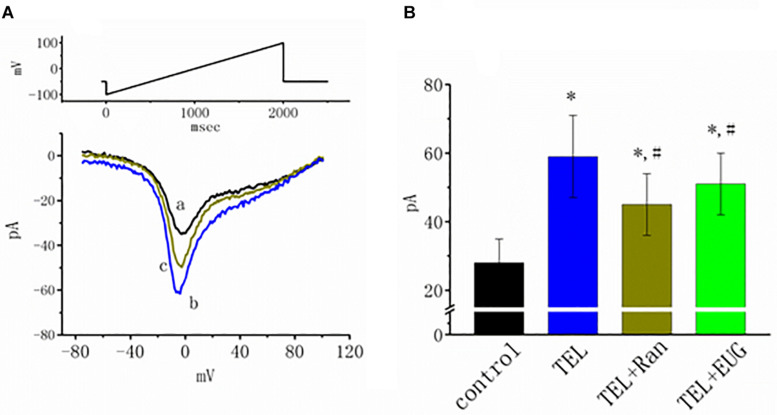
Effects of telmisartan (TEL), TEL plus ranolazine (Ran), or TEL plus eugenol (EUG) on non-inactivating *I*_*Na*_ [*I*_*Na(NI*__)_] in mHippoE-14 neurons. Each cell was voltage-clamped at −50 mV, and a 2-s long ramp pulse from −100 to +100 mV (indicated in the upper part of **A**) was applied at a rate of 0.05 Hz. **(A)** Original *I*_*Na(NI)*_ traces responding to the ramp pulse were obtained in the absence and presence of TEL and TEL plus Ran. a: control; b: TEL (3 μM); c: Tel (3 μM) plus Ran (10 μM). **(B)** Summary of the data demonstrating effects of TEL (3 μM), TEL (3 μM) plus Ran (10 μM), or TEL (3 μM) plus EUG (10 μM) on the peak amplitude of *I*_*Na(NI)*_ (mean ± SEM; *n* = 10–12 for each bar). *Significantly different from control (*p* < 0.05) and ^#^significantly different from the TEL (3 μM)-alone group (*p* < 0.05). It should be noted that either Ran or EUG can significantly reverse TEL-induced increases in the *I*_*Na(NI)*_ amplitude.

*I*_*Na(NI)*_ was previously found to be present in central neurons (Wu et al., 2009a,b). This current has been identified as playing a role in generation of epilepsy or neuropathic pain (Stafstrom, 2007; Xie et al., 2011). In another separate set of experiments, investigations were further undertaken to evaluate whether TEL has any perturbations on the amplitude of *I*_*Na(NI)*_ in response to a 2-s long-lasting ramp pulse. In this set of measurements, we bathed cells in Ca^2+^-free Tyrode’s solution containing 10 μM TEA and 0.5 mM CdCl_2_. When the tested cell was voltage clamped at −50 mV, a long-lasting ramp pulse from −100 to +100 mV was applied. The experimental observations showed that TEL enhanced *I*_*Na(NI)*_ when elicited by such a long ramp pulse (Figure 7). For example, cell exposure to TEL (3 μM) noticeably raised the peak amplitude of *I*_*Na(NI)*_ from 28 ± 7 to 59 ± 12 pA (*n* = 12, *p* < 0.05). A subsequent addition of Ran (10 μM) or EUG (10 μM) was able to attenuate TEL-perturbed stimulation of *I*_*Na(NI)*_ evoked during the long ramp pulse (Figure 7B). Similar observations were also made in the continued presence of KMUP-1 (10 μM). EUG was previously reported to be an inhibitor of *I*_*Na(NI)*_ (Huang et al., 2012). However, a further addition of angiotensin II (AT II; 200 nM) or chlorotoxin (1 μM) failed to have any effects on TEL-induced increases in *I*_*Na(NI)*_. Chlorotoxin is thought to be a blocker of Cl^–^ channels.

### Effect of TEL on Action Currents Elicited by Triangular Voltage Pulses in mHippoE-14 Neurons

**FIGURE 8:**
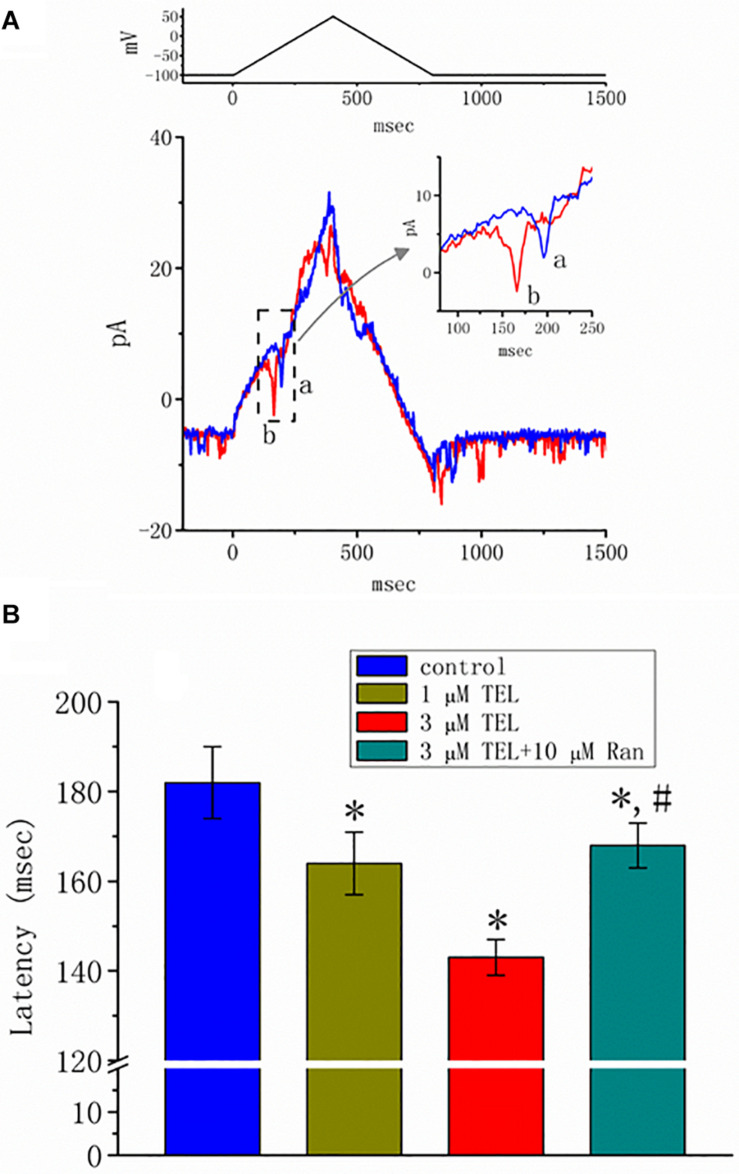
Effect of telmisartan (TEL) on action currents (ACs) in response to triangular voltage ramps. In these experiments, cell-attached current recordings were made of cells bathed in normal Tyrode’s solution containing 1.8 mM CaCl_2_. The potential was maintained at −100 mV so that, under cell-attached single-channel recordings, the membrane potential with respect to the resting potential was approximately + 30 mV. The triangular ramp pulses with a duration of 800 ms (indicated in the upper part of panel **A**) were delivered at a rate of 0.05 Hz. In **(A)**, current traces in response to triangular voltage ramps, as indicated in the upper part of the figure, were obtained in the control (a) and during cell exposure to 3 μM TEL (b). The inset indicates expanded records of action currents (ACs), which correspond to the occurrence of APs (dashed box) and appear as a brief spike in a downward direction. Noticeably, a spike appears only in the up-sloping phase but not in the down-sloping one. **(B)** Summary of data showing the effects of TEL on the latency in the generation of action currents elicited by triangular voltage ramps (mean ± SEM; *n* = 6–9 for each bar). *Significantly different from control group (*p* < 0.05) and ^#^significantly different from 3 μM TEL-alone group (*p* < 0.05).

Previous reports have disclosed that changes in the strength of late *I*_*Na*_ may affect the emergence of neuronal APs (Wu et al., 2009a,b; George et al., 2012). Questions thus arise as to whether TEL perturbs the subthreshold depolarization linked to the initiation of excitation in these cells. In this set of experiments, we bathed cells in normal Tyrode’s solution containing 1.8 mM CaCl_2_, and cell-attached current recordings were then made to measure the occurrence of ACs (Wu et al., 2009a,b). While the cell was held at −100 mV, triangular voltage pulses with a duration of 800 ms were repetitively applied. The corresponding current traces in response to this voltage-clamp protocol are illustrated in Figure 8A. Notably, the AC appears during the upsloping ramp, while the activity of large-conductance Ca^2+^-activated K^+^ channels appearing as downward deflections can be quasi-simultaneously seen at the level of −100 mV. When the cells were exposed to TEL, the latency of AC generation in response to the triangular voltage ramps was progressively shortened. Similar observations were also achieved when the cells were continually exposed to Tef (10 μM). Subsequent addition of Ran (10 μM) was noticed to reverse TEL-mediated reduction in the latency of AC generation (Figure 8B).

### Stimulatory Effects of TEL on *I*_*Na*_ in SCN5A-Expressing HEK293T Cells

**FIGURE 9:**
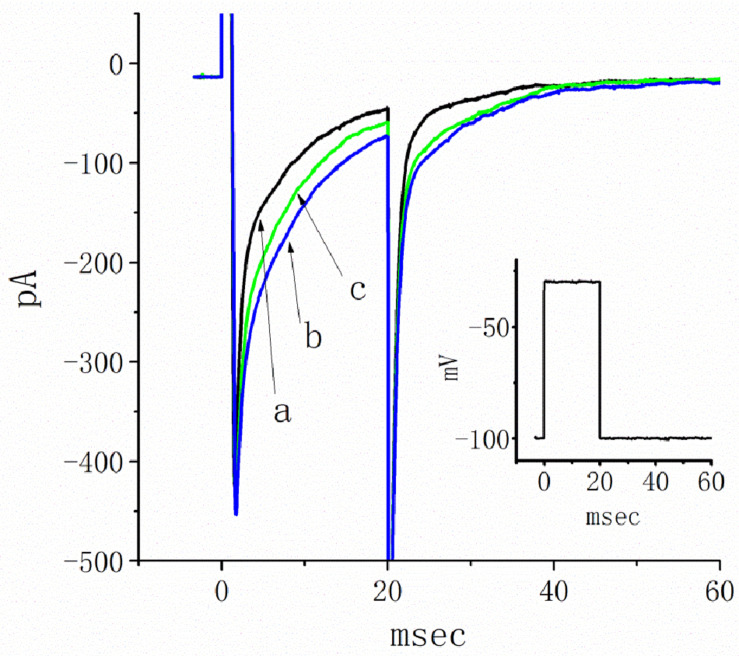
Inhibitory effects of telmisartan (TEL) on *I*_*Na*_ in HEK293T cells expressing *SCN5A*. In this set of recordings, cells were bathed in Ca^2+^-free Tyrode’s solution, and the recording pipette was filled with a Cs^+^-containing solution. The cells tested were rapidly depolarized from −100 to −30 mV for a duration of 20 ms (indicated in inset). Original current traces obtained in the absence (a) or presence of 3 μM TEL (b) and 3 μM TEL and 10 μM Ran (c).

A previous study revealed the ability of TEL to retard *I*_*Na*_ inactivation in rat ventricular myocytes (Kim et al., 2012). In a final set of experiments, we therefore evaluated whether TEL exerts any modifications on the amplitude or gating of *I*_*Na*_ in HEK293T cells transfected with *SCN5A*. Under our experimental conditions, the transfection of *SCN5A* into HEK293T cells resulted in the emergence of *I*_*Na*_. When TTX (1 μM) was applied, the *I*_*Na*_ recorded in transfected cells could be effectively suppressed. As TEL (3 μM) was applied to the bath, the peak amplitude of *I*_*Na*_ was noticeably enhanced. In addition, during exposure to TEL (3 μM), the amplitude of *I*_*Na*_ increased, and current inactivation (or relaxation) was observed to slow (Figure 9). When the cells were exposed to TEL (3 μM), the τ value of *I*_*Na*_ inactivation was significantly increased to 18.5 ± 0.3 ms from a control of 13.1 ± 2.8 ms (*n* = 6, *p* < 0.05). In the continued presence of TEL, further addition of Ran (10 μM) reversed the current inactivation time constant, as evidence by a reduction in the τ value to 14.9 ± 1.3 ms (*n* = 5, *p* < 0.05). The experimental results led us to conclude that *SCN5A*-encoded *I*_*Na*_ can indeed be expressed in HEK293T cells. The exposure to TEL is capable of diminishing both the peak amplitude and inactivation time constant of *I*_*Na*_ in HEK293T cells expressing *SCN5A*.

### Exposure to TEL Did Not Worsen Chronic Spontaneous Recurrent Seizures

**FIGURE 10:**
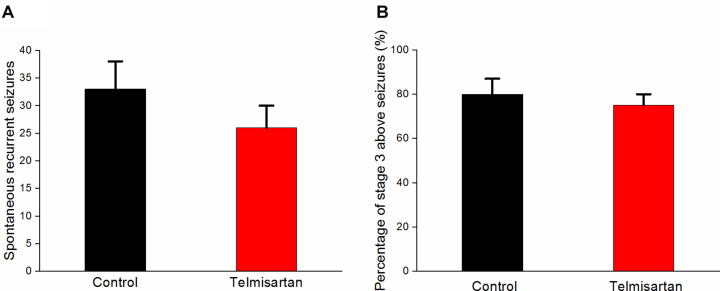
Spontaneous recurrent seizures (SRS) in both the control and telmisartan groups following status epilepticus and epileptogenesis. **(A)** Both groups showed similar numbers of SRS (*p* = 0.2) although slightly fewer seizures occurred in the Telmisartan group. **(B)** The percentage of rats with stage 3 and above seizures was similar in both groups (*p* > 0.1).

Following pilocarpine-induced status epilepticus and epileptogenesis, the control group rats and the telmisartan group exhibited a similar number of spontaneous recurrent seizures (SRS) (control: 33.8 ± 5.05 vs. telmisartan: 26.6 ± 3.50, *p* = 0.19) although there were mildly fewer seizures in the latter (Figure 10A). The percentage of rats with stage 3 and above seizure durations were similar in both the control and telmisartan groups (*p* = 0.25) (Figure 10B).

### Exposure to TEL Accentuated Hippocampal Neuronal Damage and Mossy Fiber Sprouting

**FIGURE 11:**
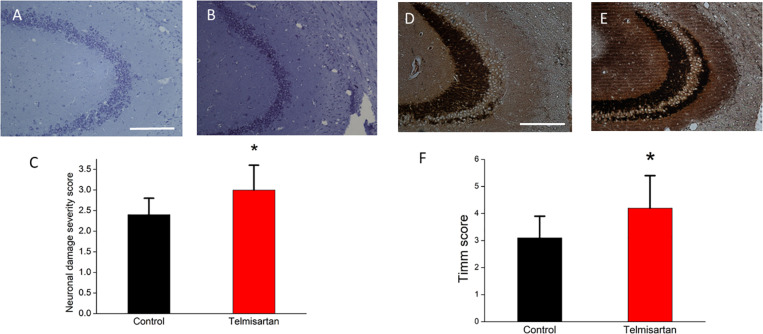
Chronic hippocampal neuronal damage and mossy fiber sprouting postpilocarpine-induced status epilepticus. **(A,B)** Cresyl violet staining showed that the telmisartan group rats had fewer neurons than the control group rats. **(C)** A blinded semiquantitative analysis showed that the hippocampal neurons in the telmisartan group rats were significantly more damaged than those in the control group rats (*p < 0.05). **(D,E)** Timm’s staining revealed that the dense mossy fiber sprouting in the hippocampal CA3 region was significantly more abundant in the telmisartan group rats with pilocarpine-induced seizures than in the control group rats, as evidenced in the Timm’s score (*p < 0.05) **(F)**.

In the chronic stage after pilocarpine-induced status epilepticus, cresyl violet staining showed that the telmisartan group rats had significantly fewer neurons (Figure 11A) than the control group rats (Figure 11B). A blinded semiquantitative analysis showed that the hippocampal neurons in the telmisartan group rats were significantly more damaged than those in the control group (damage severity score: control = 2.4 ± 0.2 vs. telmisartan = 2.8 ± 0.3, *p* < 0.05) (Figure 11C). Timm’s staining revealed that the dense mossy fiber sprouting in the hippocampal CA3 region was significantly more abundant in the telmisartan group rats with pilocarpine-induced seizures than in the control group rats (Timm’s score: control = 3.1 ± 0.8 vs. telmisartan = 4.2 ± 1.2, *p* < 0.05) (Figures 11D-F).

### Exposure to TEL Did Not Impair Cognitive Performance (Inhibitory Avoidance Task)

**FIGURE 12:**
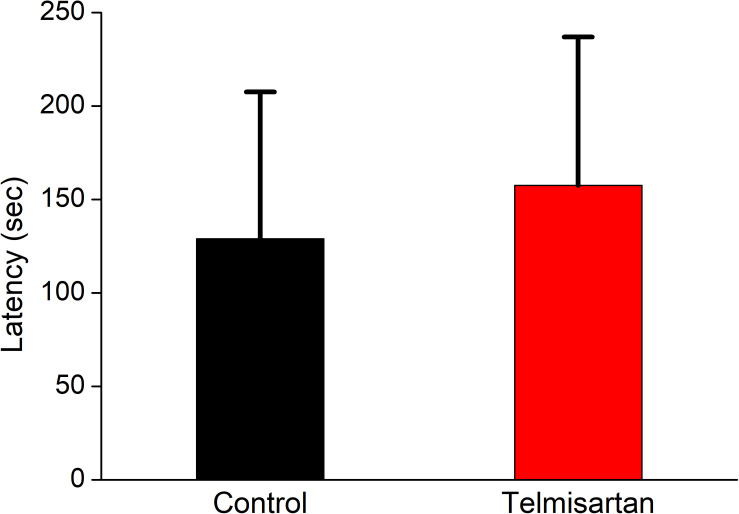
Cognitive performance (inhibitory avoidance task) following status epilepticus and epileptogenesis. After training, the latency in entering the dark compartment after training did not differ significantly between the control and the telmisartan groups although the latter tended to have relatively more retention time (*p* = 0.18).

After training, the latency when entering the dark compartment after training did not differ significantly between the control and telmisartan groups, although the latter tended to exhibit relatively more retention time (control, 128.9 ± 80 s vs. telmisartan, 157.5 ± 90s, *p* = 0.18) in these rats in the chronic stage after pilocarpine-induced status epilepticus (Figure 12).

## Discussion

The principal findings reported in this study are that TEL, a lipophilic agent, produces a stimulatory effect on *I*_*Na*_ in a concentration-, voltage-, and state-dependent fashion in mHippoE-14 neurons. TEL did not accentuate pilocarpine-induced chronic seizures and did not worsen cognitive performance; however, it indeed enhanced neuronal loss and mossy fiber sprouting in the poststatus epilepticus epileptic rats.

TEL is well recognized to display antagonistic activity toward AT1 receptors (Yusuf et al., 2008; Farsang, 2011). A number of studies have reported the presence of AT II receptors in hippocampal neurons (Premer et al., 2013; Nakagawa et al., 2017; Atanasova et al., 2018; Tashev and Ivanova, 2018). One may hence anticipate that the TEL-perturbed amplitude and kinetics of *I*_*Na*_ shown in the present study are associated with its binding to AT1 receptors and with attenuating AT II-mediated effects (Velardez et al., 2003; Wang et al., 2014). However, TEL-perturbed stimulation of *I*_*Na*_ and *I*_*Na(NI)*_ was found to be attenuated by further application of Ran or EUG but not by AT II. These results strongly led us to point out that the stimulation of Na_*V*_ channels caused by TEL is direct and appears to be unnecessarily connected with the binding to AT1 receptors, notwithstanding the possibility that these receptors are virtually expressed in the hippocampal neurons (Premer et al., 2013; Nakagawa et al., 2017; Atanasova et al., 2018; Tashev and Ivanova, 2018).

TEL-perturbed stimulation apparently is not instantaneous but rather tends to develop over time based on the openness of Na_*V*_ channels, thereby producing a resultant reduction in current inactivation. Na_*V*_1.7 was found to be a subfamily of Na_*V*_ channels functionally expressed in the hippocampal neurons (Mechaly et al., 2015). It still remains to be determined whether other isoforms of Na_*V*_ channels can be differentially subject to being stimulated by this agent or other structurally related compounds, although in the *SCN5A*-expressing HEK293T cells, TEL-stimulated effects on *I*_*Na*_ still remained effective.

In this study, TEL enhanced *I*_*Na*_, with an EC_50_ value of 0.94 μM, suggesting that this compound used for stimulation of peak *I*_*Na*_ is more potent and efficacious than either Tef or ECG (Wu et al., 2009b, 2013). The plasma TEL concentration after oral or intravenous administration of a single dose of 40 mg TEL can reach approximately 0.087 μM (44.7 ng/ml) or 2.32 μM (1,196 ng/ml), respectively (Stangier et al., 2000). By virtue of a minimal reaction scheme (Equation 2), we also found that the presence of concentration-dependent TEL slows the inactivation rate of *I*_*Na*_, with a *K*_*D*_ value of 1.04 μM. Consequently, this compound not only produced a lengthening in the inactivation time constant of *I*_*Na*_ but also shifted the *I-V* relationship of peak *I*_*Na*_ leftward and shifted the inactivation curve of *I*_*Na*_ along the voltage axis rightward. As a result, the strength of window *I*_*Na*_ would be expected to rise in the presence of TEL. This compound can thus reach the pharmacological concentrations required to be an agonist of *I*_*Na*_.

TEL has been recently noticed to exhibit an agonistic effect on PPAR-γ, which is an intranuclear receptor (Benson et al., 2004; Balaji et al., 2015). Its anti-inflammatory effects may be mediated through the activation of PPAR-γ in cells (Wang et al., 2014; Balaji et al., 2015). However, the observed response to TEL was rapid over time. Ran and EUG were also effective at attenuating TEL-stimulated *I*_*Na*_. In the continued presence of pioglitazone, an agonist of PPAR-γ, TEL-mediated stimulation of *I*_*Na*_ remained effective. Our results also showed the effectiveness of TEL in shortening the latency of AP generation in response to a triangular ramp pulse. Therefore, the stimulatory effects of this compound observed in this study are quite unlikely to be mediated through its interaction with these nuclear receptors. It is possible that the stimulatory effect of TEL on *I*_*Na*_, with an EC_50_ value of 0.94 mM, can be attributed for the most part to the results of direct interaction with Na_*V*_ channels. In addition, TEL-induced stimulation of *I*_*Na*_ was not accounted for by either binding to AT1 receptors or activation of PPAR-γ (Mogi et al., 2008). In this scenario, it is therefore possible that TEL confers protection against ischemic or traumatic brain damage possibly as a result of its activation of Na_*V*_ channels, independent of its agonistic effects on PPAR-γ activity (Mogi et al., 2008; Wang et al., 2014; Kono et al., 2015; Lin et al., 2015; Villapol et al., 2015).

A previous study showed that, in rat ventricular myocytes, TEL delays the inactivation time course of *I*_*Na*_ with minimal changes in peak *I*_*Na*_ (Kim et al., 2012). In our study, besides the slowing of the inactivation time course of *I*_*Na*_, TEL also produced a concentration-dependent increase in the amplitude of peak *I*_*Na*_, with an EC_50_ value of 0.94 mM. The reason for this discrepancy is currently unknown; however, one of the reasons could be the different Na_*V*_ isoforms (Catterall et al., 2005; Jukiè et al., 2014). The stimulatory effects of TEL observed in this study may occur at concentrations that could affect humans. Therefore, from a pharmacological standpoint, it is imperative to assess to what extent the direct actions of TEL on ionic currents observed in this study participate in its perturbing effects on different regions such as blood vessels, the pituitary gland, and neurons (Aubert et al., 2010; Du et al., 2014; Wang et al., 2014).

According to a minimal kinetic scheme (i.e., closed ↔ open ↔ inactivated) (**Equation 2**), TEL tends to have a greater affinity for both the open and open-inactivated states in Na_*V*_ channels. Since TEL is a compound with a high degree of lipophilicity and can readily cross the blood-brain barrier, there may be a relevant link between its effects on neurons or on neuroendocrine cells and its stimulatory effects on Na_*V*_ channels. In addition, in our study, because intracellular dialysis with TEL (3 μM) failed to change the amplitude and gating of *I*_*Na*_, this drug is believed to have a stimulatory effect on *I*_*Na*_ in a time-dependent fashion possibly by acting at a site that is accessible from the extracellular side of the Na_*V*_ channel. Regardless of the detailed mechanisms of TEL actions, the inherent effectiveness of TEL in activating *I*_*Na*_ and *I*_*Na(NI)*_ in different electrically excitable cells is necessarily noted with caution in relation to its increasing use as an antagonist of AT II receptors (Yusuf et al., 2008; Du et al., 2014; Łukawski et al., 2014; Wang et al., 2014).

TEL at a concentration of 3 μM failed to influence the amplitude of *I*_*Ca,L*_; however, it was effective at activating the amplitude of *I*_*Na*_ and *I*_*Na(NI)*_. Ran, EUG, and KMUP-1 were observed to reverse TEL-mediated shortening in the latency of AP generation. Such effects clearly are not explained by stimulation of *I*_*Ca,L*_. Nevertheless, further studies are needed to evaluate the extent to which TEL-perturbed stimulation of Na_*V*_ channels contribute to its pharmacological actions occurring *in vivo* (Aubert et al., 2010; Du et al., 2014; Łukawski et al., 2014).

Some earlier studies disclosed that a combination of TEL and antiepileptic drugs may lead to neurotoxic effects in animals (Łukawski et al., 2013, 2015). In the current study, TEL did not accentuate lithium–pilocarpine-induced seizures although its chronic use increased mossy fiber sprouting following status epilepticus. This relative increase in field neuronal excitability is probably associated with its stimulation of Na_*V*_ channels. As is currently known, numerous traditional and newer antiepileptic drugs are capable of attenuating Na_*V*_ channels. It is possible that the mossy fiber sprouting following status epilepticus (a major insult to the brain) develops chronically in the presence of a sodium-channel stimulator, as supported in an earlier study (Anderson et al., 2014). The lack of accentuation of seizures was supported by some previous studies comparing its various effects using several models (Łukawski et al., 2010; Pushpa et al., 2014; Łukawski and Czuczwar, 2015). In addition, it has been reported that increased sodium channel availability does not necessarily lead to increased firing rates and network excitability but rather is most sensitive to changes in the steady state activation of sodium channels (Thomas et al., 2010).

Although there was more prominent aberrant sprouting mossy fiber sprouting in the TEL group, interestingly, the group treated with TEL did not exhibit worsened cognitive impairment or inhibitory avoidance testing with a relatively longer retention time, suggesting that the enhancement of Na_*V*_-channel activity/neuronal excitability may not be always negative in individuals with epileptic seizures. In addition, an association between seizure frequency with cognitive worsening has been reported (Foster et al., 2020), which supports the observations noticed in our study. An earlier report suggested that TEL can attenuate cognitive worsening, partially through PPAR-γ activation (Mogi et al., 2008) although the sodium channel modulating mechanism appears to be independent of PPAR-γ modulation, as demonstrated in our *in vitro* study. Numerous traditional antiepileptic drugs acting on attenuation of Na_*V*_ channels have been reported to lead to worse cognitive outcomes in patients with epilepsy (Mula and Trimble, 2009), which supports our findings. The neuronal excitability and excitotoxicity in our experiments were mainly aggravated by pilocarpine modeling. Whether chronic treatment with TEL in patients with or without epileptic seizures would further enhance cognitive performance is worth further investigation. Whether clinicians need to be cautious when prescribing TEL to patients with both hypertension and epilepsy also warrants further investigation.

Collectively, the unique effects of TEL on *I*_*Na*_ in mHippoE-14 neurons are not associated with a mechanism linked to either binding to AT1 receptors or activation of PPAR-γ. Because of the importance of Na_*V*_ channels in contributing to the excitability and automaticity of hippocampal neurons, the stimulatory actions shown in this *in vitro* and *in vivo* study clearly provide novel and important insights into the pharmacomechanism for TEL effects, both in basic research and clinical practice.

## Data Availability Statement

The raw data supporting the conclusions of this article will be made available by the authors, without undue reservation.

## Ethics Statement

The animal study was reviewed and approved by Institutional Animal Care and Use Committee (IACUC).

## Author Contributions

M-CL, S-NW, and C-WH designed the experiment, analyzed the data, and wrote the manuscript. S-NW and C-WH performed the experiments and built the figures. All the authors contributed to the article and approved the submitted version.

## Conflict of Interest

The authors declare that the research was conducted in the absence of any commercial or financial relationships that could be construed as a potential conflict of interest.
